# Root Surface Electrochemical Properties Correlate with Salt Tolerance and Sodium Adsorption in *Miscanthus* Accessions: A Hydroponic Study

**DOI:** 10.3390/plants15182883

**Published:** 2026-09-21

**Authors:** Shuai Si, Yi Zhu, Yu Cheng, Hailong Lu, Huibin Li, Junqin Zong

**Affiliations:** 1 Key Laboratory of Crop Growth Regulation of Hebei Province, College of Agronomy, Hebei Agricultural University, Baoding 071000, China; ss2268936903@163.com; 2 Institute of Botany, Jiangsu Province and Chinese Academy of Sciences (Nanjing Botanical Garden Mem. Sun Yat-Sen), Nanjing 210014, China; zongjunqin@jib.ac.cn; 3 College of Ecology and Environment, Nanjing Forestry University, Nanjing 210037, China; zhuyi1027@njfu.edu.cn; 4 Marine Geological Survey of Jiangsu Province, Nanjing 210007, China; chengyu1985117@sina.com

**Keywords:** electrochemical phenotyping, *Miscanthus*, root surface charge, salt tolerance, salinity stress, sodium adsorption, zeta potential

## Abstract

Soil salinization threatens global agriculture, yet the mechanisms of salt tolerance in bioenergy crops like *Miscanthus* remain poorly understood. While root surface electrochemistry has been studied in crop species, its effect on salt tolerance among *Miscanthus* accessions remains underexplored. Therefore, this study examined whether root surface electrochemical properties are associated with differential salt tolerance and Na^+^ adsorption in eight *Miscanthus* accessions. Hydroponically grown plants were subjected to 1% NaCl for three weeks. Salt-tolerant accessions (394, 390, 422, 401) showed significantly higher relative growth parameters (81.49–89.21%) than salt-sensitive accessions (403, 1143, 01293, 11024; 61.82–66.87%; *p* < 0.05). Root surface zeta potential measurements revealed that salt-tolerant accessions carried less negative charge (ζ = −16.30 mV at pH 6.0) than salt-sensitive accessions (ζ = −30.90 mV; *p* < 0.01). Also, ATR-FTIR spectroscopy revealed a trend toward fewer ionizable functional groups, particularly carboxylates, on salt-tolerant root surfaces. Consequently, salt-sensitive accessions adsorbed significantly more Na^+^ (6847 mg kg^−1^) than salt-tolerant accessions (5163 mg kg^−1^; *p* < 0.05). Moreover, significant correlations among zeta potential, Na^+^ adsorption, and growth indices (R^2^ = 0.56, *p* < 0.5 for zeta potential vs. Na^+^ adsorption; R^2^ = 0.85, *p* < 0.01 for zeta potential vs. RRDW; R^2^ = 0.65, *p* < 0.05 for Na^+^ adsorption vs. RRDW) were consistent with the mechanistic link. This shows that fewer functional groups result in less negative charge and consequently reduced Na^+^ adsorption and enhanced salt tolerance. Therefore, salt-tolerant *Miscanthus* accessions possess fewer ionizable functional groups and less negative root surface charge, resulting in reduced Na^+^ adsorption, a mechanism not previously described in this bioenergy crop. These findings establish root surface electrochemical properties as a determinant of salt tolerance in *Miscanthus* and provide a rapid phenotyping tool for germplasm screening.

## 1. Introduction

Soil salinization currently affects more than 1.1 billion hectares globally, with saline-alkali soils making up nearly 10% of China’s total cultivated land [1,2,3,4,5]. This large-scale degradation poses severe threats to agricultural productivity and soil quality [6]. Driven by unsustainable irrigation, rising sea levels, and excessive application of chemical fertilizers, soil salinization represents one of the most significant forms of global land degradation [7]. Wang et al. projected that by 2050, soil salinization will affect approximately 50% of the irrigated land [8]. Elevated salinity disrupts plant cellular homeostasis, triggering osmotic disorder, ionic toxicity, and oxidative stress, accelerating the accumulation of reactive oxygen species and impairing plant growth and metabolism [9].

The adverse effects of soil salinization are primarily attributed to sodium ion (Na^+^) toxicity [10]. The increase in Na^+^ concentration in the rhizosphere interferes with the normal physiological processes of plants through two main mechanisms. First, Na^+^ competitively inhibits the absorption of essential nutrients, particularly potassium ions (K^+^), inducing severe nutrient deficiencies that impair cellular functions and inhibit plant growth. Second, excessive Na^+^ accumulation exerts direct toxic effects on metabolic enzymes, compromises the functional integrity of organelles, and damages the selective permeability of cell membranes [11]. Traditional research on plant salt tolerance focused on the physiological and molecular mechanisms, including the regulation of membrane transport proteins (such as HKT family proteins), vacuolar ion compartmentalization, and the synthesis of compatible osmotic regulatory substances [12,13,14]. However, the initial contact between plants and Na^+^ occurs at the root-soil interface, not at membrane transport proteins. Thus, understanding the surface electrochemistry of the roots is paramount to understanding plant tolerance to metal toxicity.

*Miscanthus*, a genus within the Poaceae (subfamily Andropogoneae), comprises approximately 17 species predominantly distributed across East Asia and Southeast Asia [14]. Among them, *Miscanthus sinensis*, *Miscanthus sacchariflorus*, *Miscanthus floridulus*, and *Miscanthus lutarioriparius* are widely distributed throughout Asia. As perennial C4 grass species, *Miscanthus* plants exhibit high photosynthetic efficiency, substantial biomass production, strong environmental adaptability, and low production cost [15,16,17]. These characteristics position *Miscanthus* as a highly promising and environmentally sustainable bioenergy crop [18,19]. Notably, *Miscanthus* species possess woody rhizomes and demonstrate greater salt tolerance than many other plant species. Nevertheless, substantial variation in salt tolerance exists among different *Miscanthus* species and accessions [20].

The plant root surface is rich in functional groups that dissociate upon protonation and deprotonation (with variation in pH), imparting a net negative charge. This surface charge plays a critical role in determining the electrostatic adsorption of cations, including Na+, onto root surfaces. According to electrochemical principles, the charged characteristics of root surfaces inevitably influence the adsorption and activity of Na^+^, thereby influencing the plant’s tolerance to Na^+^ [21,22]. Previous researchers developed a streaming potential apparatus for in situ characterization of the zeta potential of intact plant root systems [23], and successfully applied this technique to measure root surface zeta potential of crops such as rice, wheat, soybeans, and maize [24,25]. However, to date, the streaming potential method has not been employed to characterize root surface electrochemical properties of non-crop *Miscanthus* species or to investigate the relationship between these properties and salt tolerance.

The streaming potential method has been extensively validated for characterizing root surface charge properties in diverse plant species. Li et al. [23] developed and validated the method for intact rice root systems, demonstrating that measured zeta potentials were consistent with theoretical predictions based on cell wall composition and were sensitive to changes in solution pH and ionic strength. Liu et al. [26] showed that root zeta potential measured by this method correlated with Al (III) adsorption and Al tolerance in rice, while Liu et al. [24] demonstrated relationships between root surface charge and Cu/Cd adsorption in indica and japonica rice cultivars. Lu et al. [25] extended the method to 17 crop species, establishing general applicability, and Lu et al. [21] linked root surface charge to Mn (II) absorption in rice. Most recently, Lu et al. [27] validated the method for rapid screening of salt-tolerant rice genotypes, showing that root zeta potential was significantly correlated with Na^+^ adsorption and salt tolerance indices. These studies collectively demonstrate that the streaming potential method is a reliable and sensitive approach for characterizing root surface electrochemical properties and their relationship to ion adsorption and plant stress tolerance.

To address this knowledge gap, we selected eight *Miscanthus* accessions with contrasting salt tolerance as experimental materials. This study aimed to investigate three key aspects: (1) interspecific differences in salt tolerance among *Miscanthus* accessions; (2) variations in root surface charge properties and their effects on Na^+^ adsorption; and (3) correlation between root surface zeta potential and salt tolerance.

## 2. Results

### 2.1. Salt Tolerance Variation Among Miscanthus Accessions

**Figure 1:**
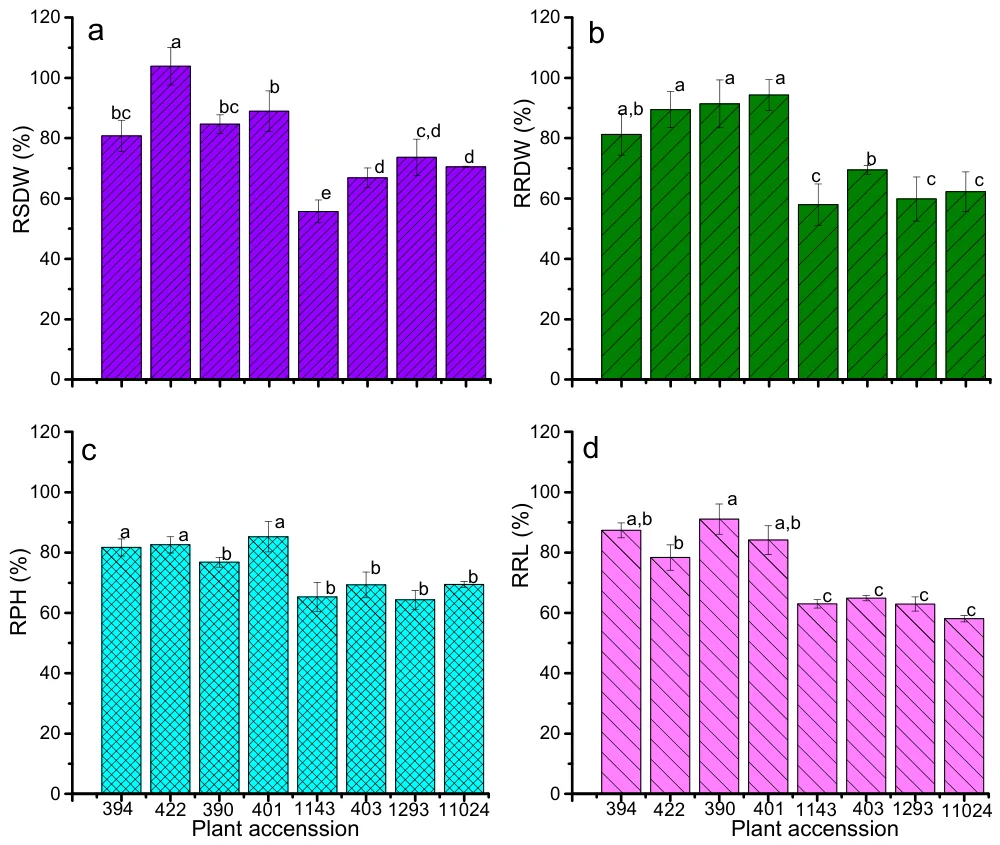
Relative shoot dry weight (**a**), relative root dry weight (**b**), relative plant height (**c**), and relative root length (**d**) of eight *Miscanthus* accessions under salt stress. Values are means ± SD (*n* = 4). Different letters indicate significant differences among accessions (*p* < 0.05, Tukey’s HSD test).

Significant differences in salt tolerance were observed among the eight *Miscanthus* accessions (Figure 1). Accessions 394, 422, 390, and 401 exhibited significantly higher relative shoot dry weight (Figure 1a), relative root dry weight (Figure 1b), relative plant height (Figure 1c), and relative root length (Figure 1d) compared to accessions 1143, 403, 01293, and 11024 (*p* < 0.05). Based on these criteria, accessions 394, 422, 390, and 401 were classified as salt-tolerant, whereas accessions 1143, 403, 01293, and 11024 were classified as salt-sensitive.

The mean values for salt-tolerant accessions were 89.21 ± 5.01% (RSW), 88.29 ± 2.86% (RRDW), 81.49 ± 1.75% (RPH), and 85.04 ± 2.73% (RRL)), whereas salt-sensitive accessions showed significantly lower values of 66.25 ± 3.78%, 61.82 ± 2.69%, 66.87 ± 1.40%, and 62.14 ± 1.44%, respectively. Among all accessions, accession 422 exhibited the maximum RSDW (103.3%), while accession 1143 showed the minimum value (55.48%) (Figure 1a).

### 2.2. Root Surface Charge and Functional Group Characteristics

**Figure 2:**
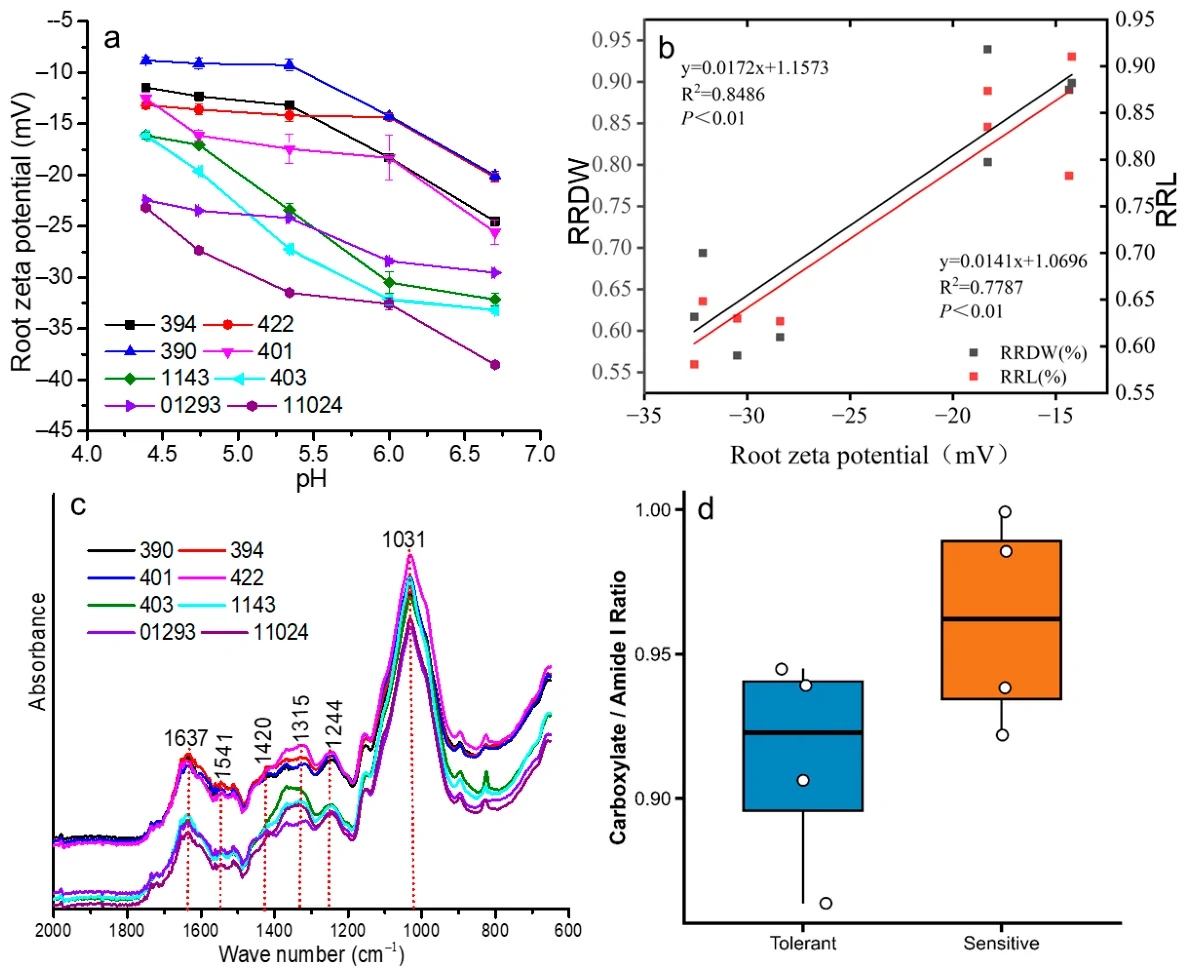
(**a**) Root surface zeta potential as a function of pH, (**b**) correlations between root zeta potential (at pH 6.0) and relative root dry weight (RRDW) and relative root length (RRL), and ATR-FTIR analysis of *Miscanthus* root surfaces: (**c**) normalized spectra (650–4000 cm^−1^) for salt-tolerant 390, 394, 401, 422) and salt-sensitive (403, 1143, 01293, 11024) accessions and (**d**) carboxylate-to-amide I ratio (1420/1637 cm^−1^). Boxplots show median, IQR, and individual points (*n* = 4 per group).

The root surface zeta potentials of all eight *Miscanthus* accessions were negative across the pH range of 4.39–6.7, with electronegativity increasing as pH increased (Figure 2a). This pH-dependent behavior indicates that negative charge density on root surfaces increases with increasing solution pH. Distinct differences in zeta potential were observed between salt-tolerant and salt-sensitive accessions. Overall, salt-sensitive accessions carried significantly more negative surface charges than salt-tolerant accessions across all pH values. Notably, the magnitude of zeta potential decline with increasing pH was substantially smaller in salt-tolerant accessions compared to salt-sensitive ones. As pH increased from 4.39 to 6.0, zeta potential decreased by 9.51 mV for salt-tolerant accessions and by 16.44 mV for salt-sensitive accessions. At pH 6.0, the absolute zeta potential of salt-sensitive accessions (30.90 ± 1.9 mV) was significantly greater than that of salt-tolerant accessions (16.30 ± 2.32 mV; *p* < 0.01, Figure 2a).

Significant negative correlations were observed between root zeta potential and both relative root dry weight (r = −0.92, *p* < 0.05) and relative root length (r = −0.88, *p* < 0.05) across the eight accessions (Figure 2b). The corresponding coefficients of determination were R^2^ = 0.85 and R^2^ = 0.78, respectively. These findings suggest that salt-tolerant *Miscanthus* accessions maintain more stable root surface charges, which may contribute to their superior salt tolerance.

ATR-FTIR spectroscopy was employed to characterize the biochemical composition of root surfaces [22,28]. The spectra revealed no obvious differences in the wavenumbers of major absorption peaks between salt-tolerant and salt-sensitive accessions (Figure 2c), indicating that similar types of functional groups were present on root surfaces of both groups. The absorption peaks at 1637 cm^−1^, 1541 cm^−1^, and 1244 cm^−1^ were assigned to the amide I band (C=O stretching and N-H bending), amide II band (N-H bending), and amide III band (C-N stretching and N-H bending) of proteins, respectively. The peak at 1420 cm^−1^ corresponded to the symmetric stretching vibration of carboxylate anions (-COO^−^). Additionally, peaks at 1315 cm^−1^ and 1031 cm^−1^ were attributed to C-OH bending vibrations of polysaccharides and cellulose, respectively [29].

After normalization to total spectral area, the relative intensities of the carboxylate peak at 1420 cm^−1^ were slightly higher in salt-sensitive accessions (14.27 ± 0.40%) than in salt-tolerant accessions (14.75 ± 0.68%), but this difference was not statistically significant (t = −1.98, df = 6.00, *p* = 0.095; Figure 2c). Similarly, the carboxylate-to-amide I ratio was higher in salt-sensitive accessions (0.961 ± 0.035) than in salt-tolerant accessions (0.913 ± 0.045, t = 1.82, df = 6.00, *p* = 0.118; Figure 2d). These results indicate that while salt-sensitive accessions tended to have a greater relative abundance of carboxylate groups on root surfaces, the differences in functional group composition between the two groups were less pronounced than initially expected based on visual inspection of the raw ATR-FTIR spectra. The lack of statistical significance may reflect the small sample size (*n* = 4 per group) and the semi-quantitative nature of ATR-FTIR analysis. Nevertheless, the trend toward higher carboxylate abundance in salt-sensitive accessions is consistent with their more negative zeta potentials and higher Na^+^ adsorption capacities.

### 2.3. Sodium Adsorption on Root Surfaces

**Figure 3:**
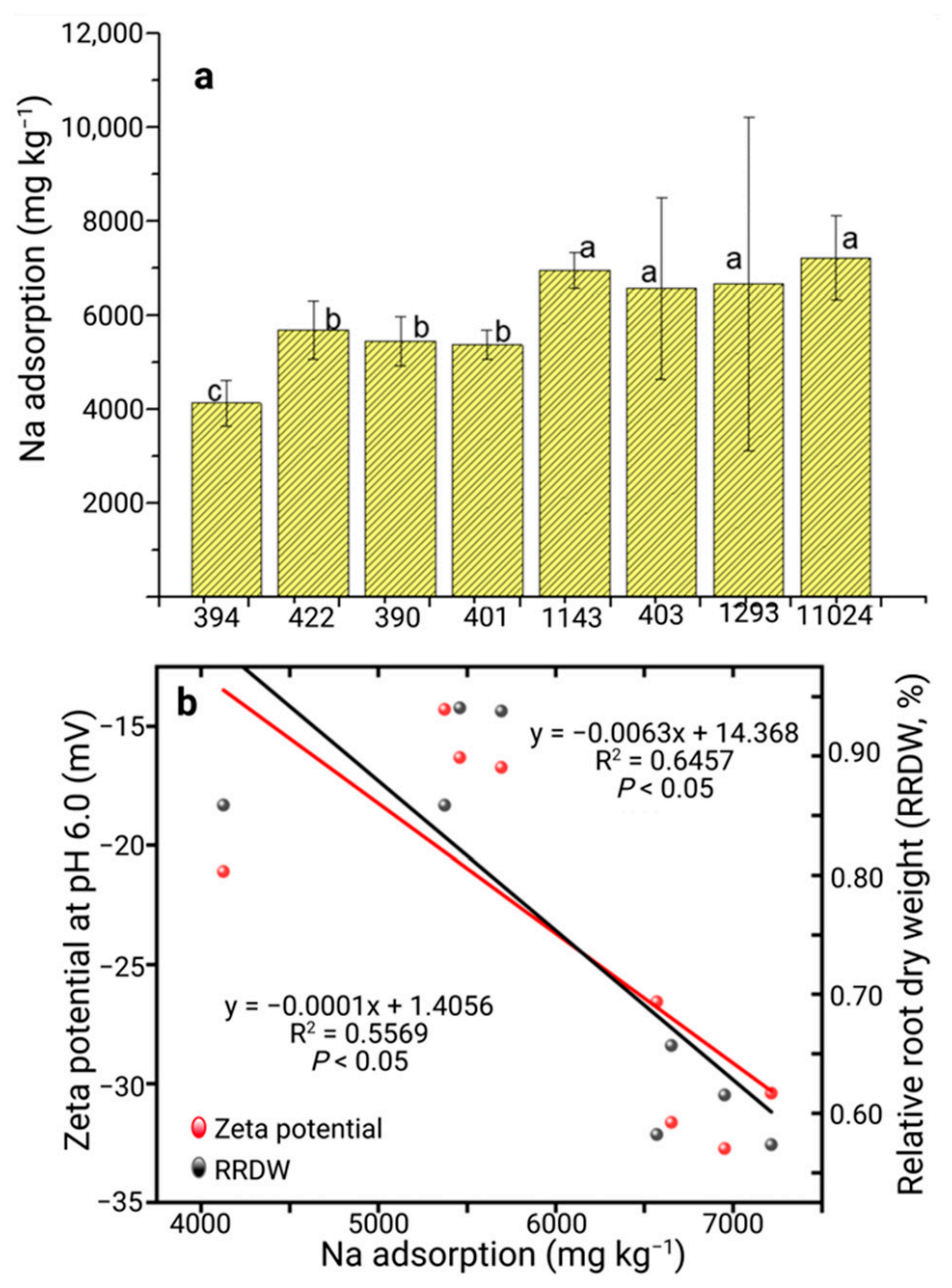
(**a**) Sodium adsorption capacity on root surfaces of eight *Miscanthus* accessions after 1.5 h treatment. Values are means ± SE (*n* = 3); (**b**) correlations among root Na^+^ adsorption capacity, zeta potential, and relative root dry weight of eight *Miscanthus* varieties. Different letters (subfigure **a**) indicate significant differences among accessions (*p* < 0.05, Tukey’s HSD test). Salt-tolerant accessions (394, 390, 422, 401) and salt-sensitive accessions (403, 1143, 01293, 11024).

Root Na^+^ adsorption capacity varied significantly among *Miscanthus* accessions (Figure 3a). The mean adsorbed Na^+^ content of salt-sensitive accessions (6847.30 ± 147.53 mg kg^−1^) was significantly higher than that of salt-tolerant accessions (5162.99 ± 352.14 mg kg^−1^; *p* < 0.05). Among all accessions, salt-tolerant accession 394 exhibited the lowest Na^+^ adsorption capacity (4126.12 mg kg^−1^), which was 23.22–27.52% lower than other salt-tolerant accessions (422, 390, 401) and 30.31–42.81% lower than salt-sensitive accessions (1143, 403, 01293, 11024).

Conversely, salt-sensitive accession 11024 showed the highest Na^+^ adsorption (7215.11 mg kg^−1^), which was 26.73–74.86% higher than all salt-tolerant accessions and 3.79–9.82% higher than other salt-sensitive accessions. These results indicate that salt-tolerant *Miscanthus* accessions adsorb less exchangeable Na^+^ on root surfaces, potentially limiting Na^+^ uptake and mitigating salt-induced damage. In addition, significant correlations were observed between root Na^+^ adsorption capacity, root zeta potential, and salt-tolerance growth indices of *Miscanthus* (Figure 3b).

### 2.4. Multivariate Analysis of Salt Tolerance Determinants

**Figure 4:**
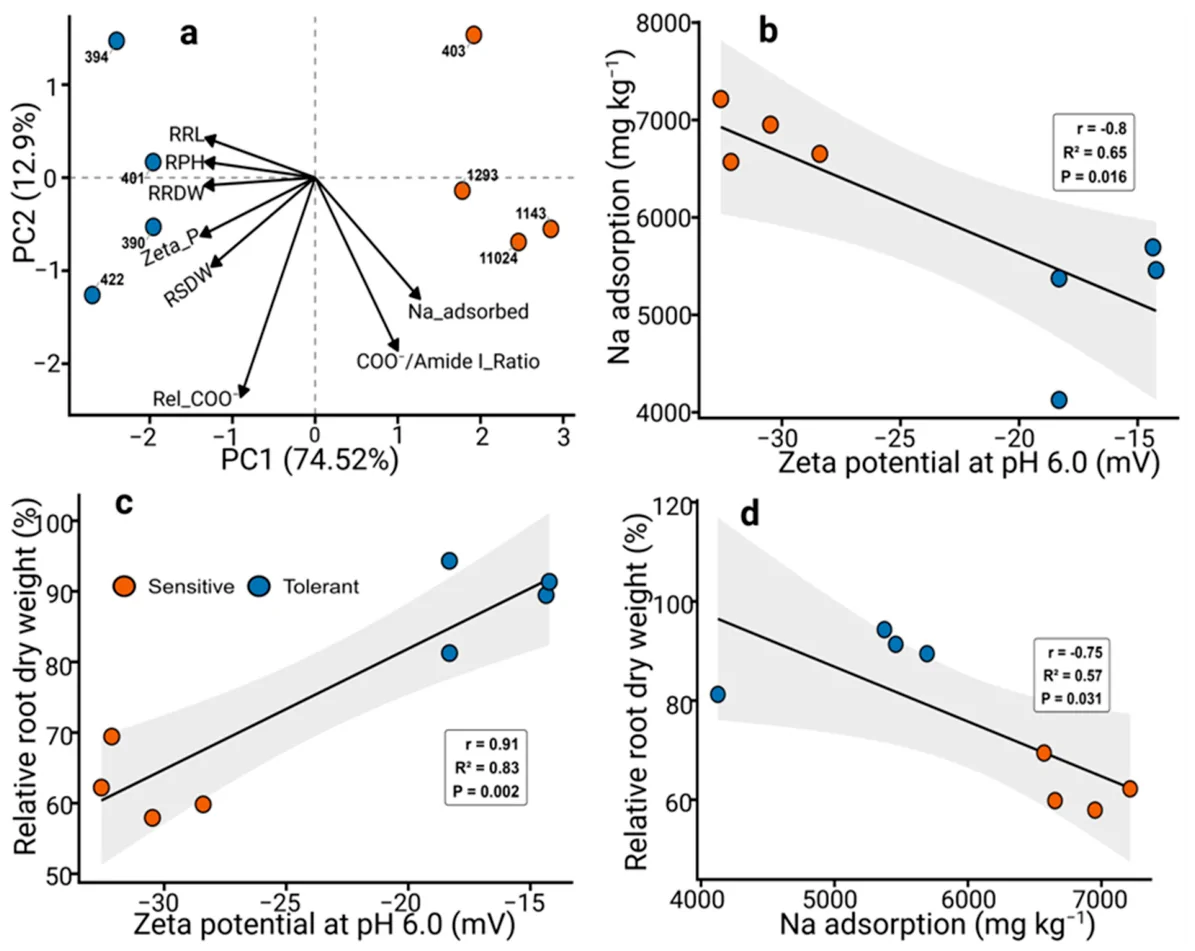
Multivariate analysis of salt tolerance determinants in eight *Miscanthus* accessions. (**a**) Principal component analysis (PCA) biplot of growth parameters (RSDW, RRDW, RPH, RRL), root surface zeta potential at pH 6.0, Na^+^ adsorption capacity, and two ATR-FTIR-derived parameters (relative carboxylate abundance and carboxylate-to-amide I ratio). Points represent individual accessions, colored by group: salt-tolerant accessions (394, 422, 390, 401) in blue and salt-sensitive accessions (1143, 403, 1293, 11024) in orange-red. Arrows represent variable loadings on PC1 and PC2. PC1 and PC2 together explained 87.42% of the total variance (PC1 = 74.52%, PC2 = 12.90%). (**b**) Simple linear regressions of Na^+^ adsorption capacity versus zeta potential, (**c**) relative root dry weight (RRDW) versus zeta potential, and (**d**) RRDW versus Na^+^ adsorption capacity. Each point represents one accession (*n* = 8), colored by group. Solid lines represent linear regression fits; shaded bands represent 95% confidence intervals. Regression statistics (r, R^2^, *p*) are shown in each panel. Significant relationships were observed for all three pairs (*p* < 0.05).

Principal component analysis (PCA) was performed to explore the integrated structure of growth, electrochemical, and ATR-FTIR data. The first two principal components (PC1 and PC2) together explained 87.42% of the total variance, with PC1 accounting for 74.52% and PC2 for 12.90% (Figure 4a). PC1 separated salt-tolerant from salt-sensitive accessions, with growth parameters (RSDW, RRDW, RPH, RRL) and zeta potential loading negatively, and Na^+^ adsorption capacity loading positively on this axis. PC2 was dominated by ATR-FTIR-derived parameters, with relative carboxylate abundance and the carboxylate-to-amide I ratio loading strongly negative. These results indicate that salt tolerance in *Miscanthus* is primarily associated with reduced Na^+^ adsorption and less negative root surface charge, with functional group composition contributing a secondary axis of variation. Linear regression analysis revealed significant relationships among root surface electrochemical properties, Na^+^ adsorption, and growth (Figure 4b–d). Zeta potential was negatively correlated with Na^+^ adsorption capacity (r = −0.80, R^2^ = 0.645, *p* = 0.016), indicating that accessions with more negative root surface charge adsorbed more Na^+^. Conversely, zeta potential was positively correlated with RRDW (r = +0.91, R^2^ = 0.827, *p* = 0.002), showing that less negative zeta potentials were associated with better growth under salt stress. Na^+^ adsorption capacity was negatively correlated with relative root dry weight (r = −0.75, R^2^ = 0.566, *p* = 0.031), confirming that increased Na^+^ adsorption impairs root growth.

Multiple regression analysis was conducted to test the hypothesized pathway linking root surface electrochemical properties, Na^+^ adsorption, and ATR-FTIR-derived functional group parameters to growth under salt stress (Table 1). The overall model was significant (F (4, 3) = 10.66, *p* = 0.040) and explained 93.4% of the variance in relative root dry weight (adjusted R^2^ = 0.847). Standardized coefficients indicated that zeta potential was the dominant predictor (standardized β = 1.585), followed by relative carboxylate abundance (β = −0.580), Na^+^ adsorption (β = 0.315), and the carboxylate-to-amide I ratio (β = −0.010). However, only zeta potential was individually significant (t = 3.76, *p* = 0.033). The non-significance of Na^+^ adsorption (*p* = 0.505) and the reversal of its coefficient sign compared to the simple regression likely reflect collinearity with zeta potential (VIF = 7.92), while the non-significant ATR-FTIR parameters are consistent with the non-significant trends observed in univariate comparisons. Sequential ANOVA confirmed that zeta potential alone accounted for the majority of explained variance (Sum Sq = 1324.76, *p* = 0.0087), while Na^+^ adsorption added negligible explanatory power (Sum Sq = 2.10, *p* = 0.823). These results position root surface zeta potential as the primary electrochemical determinant of salt tolerance in *Miscanthus*, with functional group composition contributing to a lesser extent.

## 3. Discussion

The identification of salt-tolerant *Miscanthus* accessions with favorable root surface electrochemical properties provides a basis for selecting germplasm suited to saline environments. The root surface zeta potential measurement method employed in this study offers a relatively rapid, high-throughput phenotyping approach for screening salt tolerance [27], complementing traditional physiological and molecular screening methods. Thus, root surface zeta potential measurements could provide an early, non-destructive indicator of salt tolerance potential, allowing for more efficient screening of large germplasm collections.

The results of this study demonstrate that salt-tolerant *Miscanthus* accessions possess distinct root surface electrochemical properties that reduce Na^+^ adsorption and contribute to enhanced salt tolerance (Figure 2 and Figure 3). Specifically, the growth performance of *Miscanthus* accessions under salt stress revealed clear differentiation between salt-tolerant and salt-sensitive groups (Figure 1). Salt-tolerant accessions (394, 422, 390, 401) maintained significantly higher RSDW (Figure 1a), RRDW (Figure 1b), RPH (Figure 1c), and RRL (Figure 1d) compared to salt-sensitive accessions. These findings are consistent with previous reports on salt stress responses in various plant species. Xu et al. [30] demonstrated that biomass and grain yield decreased significantly under salt stress in both tolerant and sensitive rice genotypes, though the magnitude of reduction was substantially greater in sensitive genotypes. Similarly, Nakhoda et al. [31] reported that salt-tolerant rice genotypes sustained greater biomass production with significantly higher fresh and dry weights compared to salt-sensitive genotypes. Zamani et al. [32] extended these observations to maize, finding that salt-tolerant germplasm exhibited longer root length than salt-sensitive germplasm under salt stress, a pattern that aligns with our observations in *Miscanthus*. The superior performance of salt-tolerant accessions across multiple growth parameters underscores the complex polygenic nature of salt tolerance and highlights the importance of evaluating multiple phenotypic traits when screening germplasm for stress tolerance.

Our central finding is the significant difference in root surface electrochemical properties between salt-tolerant and salt-sensitive *Miscanthus* accessions. Root surface zeta potentials were negative across all pH values tested (Figure 2a), with electronegativity increasing as pH rose, a pattern consistent with the pH-dependent dissociation of functional groups on root surfaces [23]. At pH 6.0, the absolute zeta potential of salt-sensitive accessions (30.90 mV) was nearly twice that of salt-tolerant accessions (16.30 mV). Furthermore, the magnitude of zeta potential decline with increasing pH was substantially smaller in salt-tolerant accessions (9.51 mV from pH 4.39 to 6.0) compared to salt-sensitive accessions (16.44 mV). This reduced pH sensitivity suggests that salt-tolerant *Miscanthus* accessions possess fewer pH-responsive ionizable functional groups on their root surfaces, maintaining more stable surface charge properties across varying environmental conditions. The pH range used for zeta potential measurements (4.39–6.7) does not include the alkaline pH values (pH > 8.0) typical of many saline-alkali soils. At alkaline pH, carboxylate groups are fully deprotonated, and other functional groups (e.g., hydroxyl groups on polysaccharides) may also contribute to surface charge. Additionally, alkaline pH can induce precipitation of Ca^2+^ and Mg^2+^ carbonates [33,34], altering the ionic composition of the root surface environment. Therefore, the zeta potential differences observed here may not fully capture root surface behavior under alkaline-saline conditions. Extending the pH range in future studies would improve the environmental relevance of the findings.

These findings are consistent with previous studies on root surface electrochemistry in crop species. For example, Liu et al. [24] demonstrated that root surface charge properties differed significantly between indica and japonica rice cultivars, with these differences influencing the adsorption and binding forms of Cu and Cd. Employing electrophoretic methods to measure root zeta potentials of aluminum-resistant and aluminum-sensitive rice varieties [35], it was observed that the root systems of the aluminum-sensitive variety Yangmai No. 6 carried more negative charges and adsorbed more Al (III). When investigating the effects of root surface charge and nitrogen forms on aluminum adsorption by rice roots with different aluminum tolerances, Liu et al. [26] concluded that surface charge characteristics played a critical role in determining Al sensitivity. Extending these observations to Na+ stress, Lu et al. [27] recently demonstrated that salt-tolerant rice genotypes exhibited less negative root surface charges and reduced Na^+^ adsorption compared to salt-sensitive genotypes. Our results in *Miscanthus* are in agreement with these findings, suggesting that reduced negative surface charge may represent a conserved mechanism of salt tolerance across diverse plant taxa. The consistency of this pattern across both monocot crops (rice) and perennial bioenergy grasses (*Miscanthus*) indicates that root surface electrochemical properties are fundamental determinants of plant responses to saline conditions.

The mechanistic basis for these differences likely lies in the abundance and composition of functional groups on root surfaces. ATR-FTIR analysis revealed a trend toward higher relative carboxylate abundance and higher carboxylate-to-amide I ratios (Figure 2c,d) in salt-sensitive accessions compared to salt-tolerant accessions, although these differences were not statistically significant (*p* = 0.095 and *p* = 0.118, respectively). Carboxylate groups (-COO^−^) are particularly important contributors to negative surface charge, as they undergo deprotonation at pH values above their pKa (~4–5), generating fixed negative charges that attract cations [36]. This relationship between functional group abundance, surface charge, and cation adsorption has been extensively documented in the literature. For instance, root zeta potential correlated with Mn (II) sorption across 17 crop species, establishing the general applicability of this electrochemical approach [25]. Also, it was revealed that surface charge and chemical forms of Mn (II) on rice roots influenced Mn absorption by different rice varieties, further confirming the central role of root surface electrochemistry in ion uptake [21].

Therefore, the lack of statistical significance may be due to the small sample size (*n* = 4 per group) and the semi-quantitative nature of ATR-FTIR analysis. Nevertheless, the direction of the trend is consistent with the more negative zeta potentials and higher Na^+^ adsorption capacities observed in salt-sensitive accessions. These results suggest that differences in root surface electrochemical properties may arise not only from the total abundance of carboxylate groups but also from their accessibility, local environment, or degree of deprotonation. Future studies employing quantitative methods such as cation exchange capacity (CEC) measurements, uronic acid assays, or X-ray photoelectron spectroscopy (XPS) would help resolve whether carboxylate abundance differs significantly between salt-tolerant and salt-sensitive *Miscanthus* accessions.

Na^+^ adsorption experiments provided direct evidence linking root surface electrochemical properties to salt tolerance. Salt-sensitive *Miscanthus* accessions adsorbed significantly more Na^+^ than salt-tolerant accessions, representing a 32.6% difference (Figure 3a). Among individual accessions, the range was even more striking: salt-tolerant accession 394 adsorbed 74.9% less Na^+^ compared to salt-sensitive accession 11024. This differential Na^+^ adsorption capacity has important physiological implications. By maintaining fewer negative surface charges and reducing Na^+^ adsorption, salt-tolerant *Miscanthus* accessions effectively reduce the apoplastic Na^+^ pool available for uptake and translocation. This surface-level exclusion mechanism likely operates in concert with other salt tolerance strategies. Thus, it is important to conceptually distinguish among the sequential processes that determine plant Na^+^ status under saline conditions. Peng et al. [13] demonstrated that Na^+^ compartmentalization in vacuoles contributes to salinity stress tolerance in upland cotton seedlings, while Wang et al. [12] showed that overexpression of the AtHKT1 gene improved salt tolerance in potato by regulating Na^+^ transport. In addition, Chen et al. [14] provided a comprehensive review of molecular mechanisms of salinity tolerance in rice, highlighting the roles of membrane transport proteins, including HKT family proteins, in Na^+^ exclusion and compartmentalization. Root surface Na^+^ adsorption refers to the electrostatic binding of Na^+^ to ionized functional groups (primarily carboxylates) on the apoplastic surface of root cell walls; this is a passive, physicochemical process that does not involve membrane transport. Na^+^ uptake into root tissues, by contrast, occurs via membrane transporters (e.g., HKT, NSCC, LCT1) and is driven by electrochemical gradients [12,13,14]. Hence, Na^+^ transport to shoots involves xylem loading and long-distance translocation, while intracellular Na^+^ compartmentalization refers to vacuolar sequestration via NHX antiporters and cytosolic Na^+^ extrusion via SOS1.

Hormonal regulation may influence root surface electrochemical properties. Auxin and ethylene are known to modify cell wall composition, including pectin methylation and carboxylate content, which directly affect surface charge. In rice, auxin elevates pectin content by enhancing pectin methylesterase (PME) activity, thereby increasing the availability of free carboxylate groups that contribute to cell wall negative charge [37]. Under aluminum stress, which, like Na^+^ stress, involves cation binding to root cell walls, auxin regulates cell wall modification genes through ARF10 and ARF16, and the degree of pectin methylesterification determines the Al-binding capacity of cell walls by affecting net negative charge [38,39,40]. Similarly, ethylene signaling is involved in salt-induced cell wall remodeling, with the ZAT6–LBD16 pathway regulating pectin- and xyloglucan-related gene expression and the degree of pectin methylation in root cells (ZAT6–LBD16 pathway) [41]. In spinach, salt-tolerant cultivars exhibit pectin demethylation under salinity, which alters root growth and improves salt tolerance, demonstrating that pectin characteristics directly influence root responses to salt stress [42]. Also, abscisic acid (ABA) signaling under salt stress can alter root growth and architecture, potentially changing the total surface area available for Na^+^ adsorption. ABA modulates root gravitropism and lateral root formation through PYL–PP2A signaling, which regulates auxin transport via PIN proteins under salt and osmotic stress [43]. Whether salt-tolerant *Miscanthus* accessions differ in hormone profiles or sensitivity in ways that contribute to their distinct root surface properties is an open question worthy of investigation.

Sun et al. [44] showed that salt-tolerant and salt-sensitive *M. sinensis* accessions differ in the expression of NHX1 (vacuolar Na^+^/H^+^ antiporter) and SOS1 (plasma membrane Na^+^/H^+^ antiporter), with the salt-tolerant accession showing upregulation of both genes under salinity. This suggests that transporter-mediated Na^+^ exclusion and compartmentalization operate alongside root surface exclusion mechanisms. Oxidative stress responses are also likely intertwined with root surface properties. More recently, multi-omics analyses of *M. sacchariflorus* and *M. lutarioriparius* identified transcription factor families (WRKY, bHLH, AP2/ERF, MYB, NAC) and gene modules associated with salt tolerance, including genes related to proline synthesis (P5CS1) and glutathione S-transferases (GSTZ1) for ROS detoxification [45]. Reduced Na^+^ adsorption and uptake would lower the intracellular Na^+^ load, thereby diminishing Na^+^-induced ROS production and oxidative damage to membranes, proteins, and nucleic acids. This relationship has been documented in multiple plant species under salt stress, where tissue Na^+^ and Cl^−^ accumulation is accompanied by increased H_2_O_2_ levels, lipid peroxidation, and membrane damage [46]. Salt-tolerant *Miscanthus* accessions may therefore maintain lower ROS levels not only through enhanced antioxidant systems but also through reduced Na^+^ entry at the root surface.

The above discussion reveals that salt tolerance in *Miscanthus* involves complex transcriptional networks integrating ion transport, osmotic adjustment, and oxidative stress management. Whether the accessions examined in the present study also differ in these molecular pathways, and how such differences interact with root surface electrochemical properties, warrants further investigation using transcriptomic and proteomic approaches. Moreover, experimental evidence suggests that when *M. sacchariflorus* is subjected to salt stress, it induces tissue-specific antioxidant responses, with new shoots exhibiting higher tolerance than leaves and roots, indicating that antioxidant capacity varies among tissues and developmental stages [47]. Therefore, measuring ROS accumulation, lipid peroxidation, and antioxidant enzyme activities in contrasting accessions would help clarify this relationship.

Our data demonstrate that salt-tolerant *Miscanthus* accessions exhibit reduced root surface Na^+^ adsorption, which we interpret as an early exclusion mechanism operating at the root–soil interface, upstream of, and complementary to, these membrane- and organelle-level processes. By reducing the apoplastic Na^+^ pool available for transporter-mediated uptake, this surface-level mechanism may lessen the Na^+^ load that must subsequently be managed by transport proteins and vacuolar compartmentalization. Thus, root surface electrochemistry should be viewed as a first line of defense against Na^+^ entry, not as a replacement for established physiological and molecular mechanisms of salt tolerance. Lu et al. [20] investigated the effect of salt-induced stress on the calorific value of two *Miscanthus sacchariflorus* varieties, demonstrating that salt stress affected biomass quality and energy content. Our study extends this work by identifying the root surface electrochemical basis for differential salt tolerance among *Miscanthus* accessions. Importantly, we found that salt-tolerant accessions not only maintained better growth under salt stress but also exhibited distinct root surface properties that reduced Na^+^ adsorption. Recently, the physiological response mechanisms of *Miscanthus sinensis* to salt-induced stress were deduced to provide insights into the biochemical and molecular responses of this species to salinity [19]. The findings of this study and those of other studies suggest that salt tolerance in *Miscanthus* involves multiple integrated mechanisms operating at different levels: whole-plant physiological responses, root surface electrochemical properties, and intracellular biochemical adaptations. This multi-level integration is consistent with the complex polygenic nature of salt tolerance observed in other plant species [13,14]. The significant linear correlations observed among root Na^+^ adsorption capacity, root zeta potential, and salt-tolerance growth indices (Figure 2 and Figure 3) provide quantitative support for this conclusion.

The multiple regression model explained a large proportion of the variation in root growth under salt stress (R^2^ = 0.934), with zeta potential emerging as the dominant predictor (standardized β = 1.585). Notably, the coefficient for Na^+^ adsorption was positive in the multiple regression (β = 0.315) but negative in the simple regression (r = −0.75). This sign reversal is a classic symptom of multicollinearity: zeta potential and Na^+^ adsorption are strongly correlated (VIF = 7.92–8.13, Table 1), so their individual coefficients in the multiple regression are unstable and cannot be interpreted independently. Biologically, this collinearity is expected because zeta potential and Na^+^ adsorption are mechanistically linked; a more negative surface charge drives greater Na^+^ adsorption. When both variables are included in the same model, the shared variance is attributed primarily to zeta potential, and Na^+^ adsorption contributes little additional explanatory power. This reinforces the interpretation that zeta potential is the upstream electrochemical property that governs Na^+^ adsorption and, consequently, salt tolerance. The moderate negative standardized coefficient for relative carboxylate abundance (β = −0.580) is consistent with the hypothesis that fewer carboxylate groups contribute to less negative surface charge and improved growth, although this effect was not statistically significant (*p* = 0.116) in the multiple regression.

The influence of soil chemical conditions on root surface electrochemical properties has been documented. Lu et al. [48] demonstrated that plant roots grown under acidic conditions, whether induced by NH_4_^+^ uptake in hydroponic solution or by low soil pH in pot experiments, carried lower negative surface charge and fewer functional groups than roots grown under less acidic conditions. This reduction in surface charge and functional group abundance was associated with decreased Mn (II) sorption, indicating that plants can modify their root surface properties as an adaptive mechanism to changing soil chemical environments. These findings have important implications for our study of *Miscanthus* salt tolerance. In saline-alkali soils, where pH often exceeds 8.0 and Na^+^ dominates the exchange complex, root surface properties may differ substantially from those observed in our hydroponic system (pH 6.0). Just as acidic conditions reduce root surface negative charge and functional group abundance [48], alkaline-saline conditions may also induce modifications in root surface chemistry, either through direct pH effects on functional group dissociation, through competition from Ca^2+^ and Mg^2+^ for binding sites, or through plant-mediated adaptive responses. Furthermore, the ability of plants to alter root surface properties in response to soil conditions suggests that the differences we observed among accessions may reflect both constitutive (genetic) differences and inducible (plastic) responses to salt stress.

While this study provides evidence for the role of root surface electrochemical properties in *Miscanthus* salt tolerance, several limitations are acknowledged. The use of dried roots for zeta potential measurement is a significant limitation given that we frame root surface electrochemistry as an in vivo mechanism operating at the living root–soil interface. While drying may preserve the relative differences among accessions, absolute zeta potential values should be interpreted with caution. In situ streaming potential measurements on intact, living roots, as developed by Li et al. [23], would provide a more accurate assessment and should be prioritized in future work. Additionally, complementary approaches such as electrophoretic light scattering on isolated root cell walls or microelectrode-based surface potential measurements could help bridge the gap between dried-root measurements and living-root conditions. Future studies employing in situ streaming potential measurements on intact, living root systems would provide a more accurate assessment of root surface properties under physiological conditions.

Another limitation of this study is that tissue Na^+^ and K^+^ concentrations were not measured. Our focus was on characterizing the root surface electrochemical properties that govern the initial interaction between roots and Na^+^ in the rhizosphere, rather than on whole-plant ion accumulation. However, tissue ion concentrations, particularly the shoot Na^+^/K^+^ ratio, are key physiological indicators of salt tolerance in glycophytes [11], and measuring them would provide a direct bridge between root surface Na^+^ adsorption and whole-plant ion homeostasis. Without these data, we cannot confirm that reduced surface adsorption translates into reduced Na^+^ uptake and accumulation in salt-tolerant accessions. Nevertheless, it is possible that differences in transporter-mediated Na^+^ exclusion, vacuolar compartmentalization, or xylem loading also contribute to the observed growth differences among accessions. Future studies should measure shoot and root Na^+^ and K^+^ concentrations in salt-treated plants to determine whether the accessions with less negative root surface charge and lower Na^+^ adsorption also maintain lower tissue Na^+^ concentrations and higher K^+^/Na^+^ ratios. Such data would strengthen the causal chain linking root surface electrochemistry to whole-plant salt tolerance.

This study used juvenile plants (20–30 cm in height) grown under hydroponic conditions, and it is unknown whether the observed relationships between root surface electrochemical properties and salt tolerance hold for mature plants. Root surface properties can change with plant age due to alterations in cell wall composition, suberin deposition, and rhizosphere microbial associations [36]. In perennial grasses such as *Miscanthus*, root systems undergo continuous growth and senescence, and the proportion of young versus old roots may influence bulk surface charge measurements. Future studies should compare root surface electrochemical properties across developmental stages (seedling, juvenile, mature, and regrowth stages) and determine whether the ranking of salt-tolerant versus salt-sensitive accessions is consistent. Additionally, field-grown mature plants may exhibit different surface charge properties due to interactions with soil particles, organic matter, and microbial biofilms, which cannot be captured in hydroponic systems.

The experiments were conducted under controlled hydroponic conditions, which allow for precise control over ion concentrations and eliminate confounding soil factors. To address these limitations, future studies should validate our findings under field conditions and in soil-based systems. Specifically, paired experiments comparing hydroponically grown and soil-grown *Miscanthus* accessions would determine whether the root surface electrochemical differences observed here persist in the presence of soil solids, organic matter, and microbial communities. Soil solution composition, particularly the concentrations of Ca^2+^, Mg^2+^, and K^+^, may compete with Na^+^ for binding sites on root surfaces and alter the relationship between zeta potential and Na^+^ adsorption. Additionally, soil pH buffering and the presence of clay minerals and organic colloids may modify root surface charge properties through adsorption of dissolved organic matter and metal oxides. Field trials across multiple sites with naturally saline-alkali soils would be necessary to confirm whether the accessions identified here as salt-tolerant maintain their superior performance under realistic stress conditions. Such validation is essential before root surface zeta potential can be adopted as a reliable phenotyping tool for breeding programs targeting saline-alkali lands.

## 4. Materials and Methods

### 4.1. Plant Cultivation, Growth Parameters, and Sorption-Desorption Experiment

The eight *Miscanthus* accessions selected as experimental materials are described in Table 2. Seeds were multiplied under uniform greenhouse conditions for one generation prior to experimentation to minimize maternal environmental effects. The selected seeds were soaked in distilled water for 4 h and then placed on moist filter paper in Petri dishes for dark germination. When the plants grew to 4 cm in height, three seedlings were grouped and wrapped with planting cotton (with a diameter of 2.5 cm and a height of 2 cm) and inserted into the small holes (with a diameter of 1.5 cm) of a 16-hole custom-made ABS plastic board. Each board accommodated four *Miscanthus* accessions (*n* = 4), with four holes per accession, and each hole contained three seedlings. The experiment used a completely randomized design with four replicate boards per accession, yielding 16 replicate holes (48 individual plants) per accession. For growth parameter measurements, all plants were measured, and board-level means were calculated from the four replicate boards, giving *n* = 4 biological replicates per accession for statistical analysis.

The plastic boards were placed in a 5 L black square container filled with hydroponic nutrient solution and continuously aerated. The nutrient solution composition was as follows (mM): 2.5 Ca(NO_3_)_2_·4 H_2_O, 2.5 KNO_3_, 1.0 MgSO_4_ 7·H_2_O, 0.5 NH_4_H_2_PO_4_, 0.5 KCl, µM: 46 H_3_BO_3_, 9 MnCl_2_·4 H_2_O, 0.8 ZnSO_4_·7 H_2_O, 0.3 CuSO_4_·5 H_2_O, 0.1 (NH_4_)_6_Mo_7_O_24_, 100 Fe (Fe-EDTA). The nutrient solution pH was adjusted to 6.0 ± 0.2 and renewed every 7 d with continuous aeration throughout the cultivation period. When the plants reached 20–30 cm in height, salt stress was applied by adding NaCl to a final concentration of 1% (*w*/*v*). Control plants were maintained in a NaCl-free nutrient solution. After 3 weeks of treatment, shoots and roots were harvested for subsequent experiments.

Shoots and roots were rinsed three times with deionized water and immediately separated. Samples were deactivated at 105 °C for 30 min, oven-dried at 80 °C to constant weight, and then weighed. The following relative growth indices were calculated: (i) Relative shoot dry weight (RSDW) = salt treatment shoot dry weight/control shoot dry weight × 100%; (ii) Relative root dry weight (RRDW) = salt treatment root dry weight/control root dry weight × 100%; (iii) Relative plant height (RPH) = salt treatment plant height/control total plant height × 100%; and (iv) Relative root length (RRL) = salt treatment root length/control root length × 100%.

Prior to the assay, roots from the salt-free control group were soaked in deionized water for 12 h, with deionized water renewed every 4 h to eliminate residual ions on root surfaces. Afterwards, the roots were transferred into 300 mL of 1% NaCl solution, and Na^+^ adsorption was performed under magnetic stirring for 1.5 h. Upon completion of adsorption, roots were rinsed three times with deionized water and transferred into 200 mL of 0.1 mol L^−1^ KNO_3_ solution for 0.5 h of desorption to extract exchangeable Na^+^ adsorbed on root surfaces. This design was chosen to characterize baseline differences in root surface electrochemical properties among accessions, independent of stress-induced modifications. However, root surface charge properties may change under salt stress due to alterations in cell wall composition, pectin methylation, and suberin deposition [36]. Therefore, the Na^+^ adsorption capacities reported here represent intrinsic differences among accessions rather than stress-acclimated responses. Future studies should measure Na^+^ adsorption on roots harvested from salt-stressed plants at multiple time points to capture the temporal dynamics of surface charge modification during salt acclimation. After desorption, root samples were collected, deactivated at 105 °C for 30 min, oven-dried at 80 °C, and weighed. The Na^+^ concentration in the extracting solution was measured by flame photometer. Three replicates were performed for each treatment.

### 4.2. Root Surface Zeta Potential and Functional Group Determination

Root surface zeta potential was measured for all eight accessions using the streaming potential method as described by Li et al. [23]. Dried intact root systems were cut into segments of 2.0 ± 0.2 cm, thoroughly mixed, and loaded into the sample cell. We acknowledge that zeta potential measurements on dried root segments may not fully represent the in vivo condition of living roots. Drying can alter the conformation and accessibility of cell wall functional groups, potentially affecting surface charge measurements. However, dried roots were used to ensure methodological consistency with prior studies [23,24,25] and to eliminate variability associated with root metabolic activity during measurement.

A total of 1.0 L NaCl electrolyte solution (conductivity = 80 ± 2 μS/cm) was poured into the storage tank and equilibrated for 1.5 h. Afterwards, the solution was replaced with 1.0 L of fresh NaCl electrolyte solution, followed by another 5 min equilibration before measurement. Five pH gradients (pH 6.7, 6.0, 5.34, 4.74, 4.39) were used to evaluate the root zeta potential [23]. The pH range of 4.39–6.7 was selected to span the typical apoplastic pH range of plant roots (approximately 5.0–6.5) and to encompass the pKa of carboxylate groups, which are the dominant contributors to root surface negative charge. This range is consistent with prior studies using the streaming potential method [23,36]. However, we acknowledge that saline-alkali soils often have pH values above 8.0, and the absence of near-neutral and alkaline pH points is a limitation. Ag/AgCl and Pt electrodes were employed to respectively measure the flow potential (ΔE) and conductivity (κ) at both ends of the sample cell. Afterwards, the zeta potential of the root surface was determined from Equation (1) [49].

(1) $$\zeta =\frac{\Delta E}{\Delta P}\frac{\mu }{\varepsilon \varepsilon_{0}}k$$

where ζ represents the zeta potential (mV). ΔE and ΔP denote the streaming potential (mV) and liquid pressure difference (Pa), respectively. μ, ε, ε_0_, and κ designate the solution viscosity (Pa·s), dielectric constant of electrolyte solution (F/m), vacuum permittivity (F/m), and solution conductivity (S/m), respectively.

### 4.3. Spectroscopic Characterization of Roots

After washing and drying the harvested plant roots, the infrared spectra of the root surfaces were measured using a Nicolet iS10 infrared spectrometer (Nicolet Analytical Instruments, Madison, WI, USA) equipped with a diamond ATR probe. Spectra were recorded over the range of 650–4000 cm^−1^ at a resolution of 4 cm^−1^, with 32 scans collected per sample.

### 4.4. Sodium Adsorption and Desorption Assay

Prior to peak intensity comparison, spectra were normalized to the total area under the 650–4000 cm^−1^ region using the trapezoidal rule to account for variations in sample contact pressure, root thickness, and water content. This normalization allows semi-quantitative comparison of relative functional group abundance among accessions. Peak intensities at 1637, 1541, 1420, 1315, and 1031 cm^−1^ were extracted and expressed as percentages of the sum of these five peaks. Additionally, the ratio of carboxylate (1420 cm^−1^) to amide I (1637 cm^−1^) was calculated as an internal indicator of carboxylate abundance relative to protein content. Independent-samples *t*-tests were used to compare relative peak intensities and carboxylate-to-amide I ratios between salt-tolerant and salt-sensitive groups. Significance was set at *p* < 0.05.

### 4.5. Statistical Analysis

More statistical analyses were performed using SPSS 27.0 (IBM, Armonk, NY, USA). One-way analysis of variance (ANOVA) was employed to detect differences in measured parameters among accessions, followed by Tukey’s HSD post hoc test for multiple comparisons when appropriate. Independent-samples *t*-tests were used to compare indicators between salt-tolerant and salt-sensitive groups. Pearson bivariate correlation analysis was conducted to explore relationships among variables. Significance was set at *p* < 0.05.

In addition to univariate analyses, multivariate statistical methods were employed to explore the integrated structure of the dataset. Principal component analysis (PCA) was performed on the correlation matrix of growth parameters (RSDW, RRDW, RPH, RRL), root surface zeta potential (at pH 6.0), root Na^+^ adsorption capacity, and two ATR-FTIR-derived parameters: relative carboxylate abundance (1420 cm^−1^ as a percentage of five major peaks) and the carboxylate-to-amide I ratio (1420/1637 cm^−1^). These two parameters were selected to avoid redundancy among the five relative peak percentages, which sum to 100%, and to introduce compositional closure. Linear regression analysis was used to quantify the relationships between zeta potential and Na^+^ adsorption, zeta potential and growth indices, and Na^+^ adsorption and growth indices. To test the hypothesized pathway linking functional group abundance to growth via zeta potential and Na^+^ adsorption, multiple regression analysis was conducted with growth indices as dependent variables and zeta potential, Na^+^ adsorption, and the two ATR-FTIR parameters as predictors. All multivariate analyses were performed using R version 4.3.1 (R Foundation for Statistical Computing, Vienna, Austria). PCA was conducted using the prcomp() function with standardized variables. Linear and multiple regression analyses were performed using the lm() function. Multicollinearity was assessed using variance inflation factors (VIF). Figures were generated using the ggplot2, factoextra, and patchwork packages.

## 5. Conclusions

This study demonstrates significant differences in root surface electrochemical properties and Na^+^ adsorption capacity among *Miscanthus* accessions with contrasting salt tolerance. Salt-tolerant accessions possess less negative surface charge and reduced Na^+^ adsorption compared to salt-sensitive accessions. ATR-FTIR analysis revealed a consistent but non-significant trend toward fewer carboxylate groups on salt-tolerant root surfaces, suggesting that other factors beyond total functional group abundance may contribute to the observed differences in surface charge. The decreased functional group abundance, lower negative surface charge, and diminished Na^+^ adsorption capacity of salt-tolerant accession roots likely contribute to reduced Na^+^ uptake and accumulation. These findings provide a theoretical foundation for screening and breeding salt-tolerant *Miscanthus* germplasm for cultivation on saline-alkali lands and offer a rapid electrochemical phenotyping approach for evaluating salt tolerance in this economically important bioenergy crop. While this study establishes root surface electrochemical properties as a determinant of Na^+^ adsorption in *Miscanthus*, the absence of tissue Na^+^ and K^+^ data limits our ability to link surface adsorption directly to whole-plant ion homeostasis. Future work integrating root surface characterization with tissue ion analysis will be essential to fully elucidate the mechanisms of salt tolerance in this bioenergy crop.

A key limitation of this study is the relatively small number of accessions examined (*n* = 8). While these accessions were selected to represent contrasting salt tolerance phenotypes, they capture only a fraction of the genetic diversity within the genus *Miscanthus*, which comprises approximately 17 species and numerous ecotypes across East Asia [50,51]. The strong correlations observed between root surface electrochemical properties and salt tolerance are promising, but they require validation across a larger, more diverse panel of accessions. Future studies should include a minimum of 30–50 accessions spanning multiple species (e.g., *M. sinensis*, *M. sacchariflorus*, *M. floridulus*, *M. lutarioriparius*) and ecotypes from contrasting climates and soil types. Such a panel would allow for robust statistical analyses, including genome-wide association studies to identify genetic loci underlying root surface charge variation. Until such validation is performed, the conclusions of this study should be considered preliminary and specific to the accessions examined.

## Figures and Tables

**Table 1:** Multiple regression analysis of root surface electrochemical properties, Na^+^ adsorption, and ATR-FTIR-derived functional group parameters as predictors of relative root dry weight (RRDW) in eight *Miscanthus* accessions under salt stress.

Predictor	Estimate	SE	Standardized β	t	*p*	VIF
(Intercept)	435.59	161.20	-	2.70	0.074	-
Zeta potential (mV)	2.98	0.79	1.585	3.76	0.033	8.13
Na^+^ adsorption (mg kg^−1^)	0.0046	0.0061	0.315	0.76	0.505	7.92
Relative carboxylate (%)	−21.65	9.87	−0.580	−2.20	0.116	3.19
Carboxylate/amide I ratio	−3.41	95.01	−0.010	−0.04	0.974	3.31
Model summary						
R^2^	0.934					
Adjusted R^2^	0.847					
F-statistic	10.66 (df = 4, 3)				0.040	
Residual SE	5.92					

Note: Dependent variable = relative root dry weight (RRDW, %). *n* = 8 accessions. Standardized β = standardized regression coefficient. VIF = variance inflation factor. Bold values indicate statistical significance at *p* < 0.05. Collinearity between zeta potential and Na^+^ adsorption (VIF > 5) should be noted when interpreting individual coefficients. SE = standard error.

**Table 2:** Source, collection origin, species identity, and registry information of the eight *Miscanthus* accessions used in this study.

Number	Source Location	Specific Name	Latitude	Longitude	Altitude
394	Wuhu city, Anhui province	*M. sacchariflorus*	31.2176	118.209	10
422	Yangzhou city, Jiangsu province	*M. sacchariflorus*	32.1414	119.227	−11
390	Dongying city, Shandong province	*M. sacchariflorus*	37.4592	119.041	−1
401	Wuhu city, Anhui province	*M. sacchariflorus*	31.1692	118.221	2
1143	Hanshou city, Hunan province	*M. sacchariflorus*	29.0442	111.593	26
403	Nanjing city, Jiangsu province	*M. sacchariflorus*	32.0938	118.714	13
11024	Dongtai city, Jiangsu province	*M. sacchariflorus*	32.57	120.5	1
01293	Tongling city, Jiangsu province	*M. floridulus*	30.58	117.59	13

## Data Availability

The Dataset is available on request from the authors.

## References

[1] Shokri N., Hassani A., Sahimi M. (2024). Multi-Scale Soil Salinization Dynamics from Global to Pore Scale: A Review. Rev. Geophys..

[2] Wang B., Li X., Gao Z., Jia Z., Liang Z. (2025). A 40-year dataset of soil salinity dynamics (1985–2024) at 100 m resolution in the Western Songnen Plain, China. Sci. Data.

[3] Han Q., Wang S., Liu B., Zhou J., Liu C. (2025). Spatial heterogeneity and driving factors of soil salinity in the lowland area of North China plain. Vadose Zone J..

[4] Haonan Q., Shihong Y., Guangmei W., Jie Z., Xiaoling L., Yi X., Shide D., Zewei J., Hanwen L., Mouazen A.M. (2026). Evolutionary patterns of soil salinization and saline-alkali land types in the Yellow River Delta: A spatiotemporal analysis from 2002 to 2022. Soil Tillage Res..

[5] Li J., Pu L., Han M., Zhu M., Zhang R., Xiang Y. (2014). Soil salinization research in China: Advances and prospects. J. Geogr. Sci..

[6] Hassani A., Azapagic A., Shokri N. (2021). Global predictions of primary soil salinization under changing climate in the 21st century. Nat. Commun..

[7] Zhang Q., Shen X. (2023). Progress of Water and Salt Transport in Saline Lands and Hydrus Model: A Review. Acad. J. Sci. Technol..

[8] Wang X., Chen Z., Sui N. (2024). Sensitivity and responses of chloroplasts to salt stress in plants. Front. Plant Sci..

[9] Kumari S., Mahajan M., Naseem K., Kaur P., Chaudhary A.A., Bendif H., Khan M.I.R. (2026). Dopamine regulates nitric oxide biosynthesis, optimizes carbon utilization, and maintains potassium retention to support wheat productivity under salt stress. Plant Physiol. Biochem..

[10] Zhang J.-L., Flowers T.J., Wang S.-M. (2010). Mechanisms of sodium uptake by roots of higher plants. Plant Soil.

[11] Maathuis F.J.M., Amtmann A. (1999). K^+^ nutrition and Na^+^ toxicity: The basis of cellular K^+^/Na^+^ ratios. Ann. Bot..

[12] Wang L., Liu Y., Li D., Feng S., Yang J., Zhang J., Zhang J., Wang D., Gan Y. (2019). Improving salt tolerance in potato through overexpression of AtHKT1 gene. BMC Plant Biol..

[13] Peng Z., He S., Sun J., Pan Z., Gong W., Lu Y., Du X. (2016). Na+ compartmentalization related to salinity stress tolerance in upland cotton (*Gossypium hirsutum*) seedlings. Sci. Rep..

[14] Chen T., Shabala S., Niu Y., Chen Z.-H., Shabala L., Meinke H., Venkataraman G., Pareek A., Xu J., Zhou M. (2021). Molecular mechanisms of salinity tolerance in rice. Crop J..

[15] Wang J., Duan X., Wang Y., Sheng J. (2022). Transcriptomic and physiological analyses of *Miscanthus lutarioriparius* in response to plumbum stress. Ind. Crops Prod..

[16] Yang S., Xue S., Kang W., Qian Z., Yi Z. (2019). Genetic diversity and population structure of *Miscanthus lutarioriparius*, an endemic plant of China. PLoS ONE.

[17] Sa M., Zhang B., Zhu S. (2021). *Miscanthus*: Beyond its Use as an Energy Crop. Bioresources.

[18] Heaton E.A., Dohleman F.G., Long S.P. (2008). Meeting US biofuel goals with less land: The potential of *Miscanthus*. Glob. Change Biol..

[19] Lu H.-L., Li L., Nkoh J.N., Li J.-J., Wang H.-R., Li X.-H., Hao D.-L., Zong J.-Q. (2025). Deciphering the physiological response mechanisms of *Miscanthus sinensis* to salt-induced stress. Biomass Bioenergy.

[20] Lu H., Li L., Chen J., Nkoh J.N., Hao D., Li J., Wang J., Li D., Liu J., Guo H. (2024). Effect of Salt-Induced Stress on the Calorific Value of Two *Miscanthus sacchariflorus* (Amur Silvergrass) Varieties. Agronomy.

[21] Lu H., Nkoh J.N., Biswash M.R., Hua H., Dong G., Li J., Xu R. (2021). Effects of surface charge and chemical forms of manganese(II) on rice roots on manganese absorption by different rice varieties. Ecotoxicol. Environ. Saf..

[22] Sharifi M., Khoshgoftarmanesh A.H., Hadadzadeh H. (2016). Changes in the chemical properties and swelling coefficient of alfalfa root cell walls in the presence of toluene as a toxic agent. Environ. Sci. Pollut. Res..

[23] Li Z., Liu Y., Zheng Y., Xu R. (2015). Zeta potential at the root surfaces of rice characterized by streaming potential measurements. Plant Soil.

[24] Liu Z.-D., Zhou Q., Hong Z.-N., Xu R.-K. (2017). Effects of Surface Charge and Functional Groups on the Adsorption and Binding Forms of Cu and Cd on Roots of indica and japonica Rice Cultivars. Front Plant Sci..

[25] Lu H., Liu Z., Zhou Q., Xu R. (2018). Zeta potential of roots determined by the streaming potential method in relation to their Mn(II) sorption in 17 crops. Plant Soil.

[26] Liu Z.-D., Wang H., Xu R. (2016). The effects of root surface charge and nitrogen forms on the adsorption of aluminum ions by the roots of rice with different aluminum tolerances. Plant Soil.

[27] Lu H., Xiang X., Si S., Nkoh J.N., Li L., Hao D., Jiang C., Liu Q., Zong J., Xu R. (2026). Hydroponic electrochemical phenotyping of root surfaces for rapid screening of salt-tolerant rice (*Oryza sativa* L.). Ind. Crops Prod..

[28] Kacuráková M. (2000). FT-IR study of plant cell wall model compounds: Pectic polysaccharides and hemicelluloses. Carbohydr. Polym..

[29] Durak T., Depciuch J. (2020). Effect of plant sample preparation and measuring methods on ATR-FTIR spectra results. Environ. Exp. Bot..

[30] Xu Y., Bu W., Xu Y., Fei H., Zhu Y., Ahmad I., Nimir N.E.A., Zhou G., Zhu G. (2024). Effects of Salt Stress on Physiological and Agronomic Traits of Rice Genotypes with Contrasting Salt Tolerance. Plants.

[31] Nakhoda B., Leung H., Mendioro M.S., Mohammadi-nejad G., Ismail A.M. (2012). Isolation, characterization, and field evaluation of rice (*Oryza sativa* L., Var. IR64) mutants with altered responses to salt stress. Field Crops Res..

[32] Zamani E., Bakhtari B., Razi H., Hildebrand D., Moghadam A., Alemzadeh A. (2024). Comparative morphological, physiological, and biochemical traits in sensitive and tolerant maize genotypes in response to salinity and Pb stress. Sci. Rep..

[33] Sparks D.L., Singh B., Siebecker M.G. (2024). An Introduction to Environmental Soil Chemistry. Environmental Soil Chemistry.

[34] Kopittke P.M. (2012). Interactions between Ca, Mg, Na and K: Alleviation of toxicity in saline solutions. Plant Soil.

[35] Wang W., Zhao X.Q., Chen R.F., Dong X.Y., Lan P., Ma J.F., Shen R.F. (2015). Altered cell wall properties are responsible for ammonium-reduced aluminium accumulation in rice roots. Plant Cell Environ..

[36] Zhou Q., Liu Z., Liu Y., Jiang J., Xu R. (2016). Relative abundance of chemical forms of Cu(II) and Cd(II) on soybean roots as influenced by pH, cations and organic acids. Sci. Rep..

[37] Huang J., Wu Q., Jing H.K., Shen R.F., Zhu X.F. (2022). Auxin facilitates cell wall phosphorus reutilization in a nitric oxide-ethylene dependent manner in phosphorus deficient rice (*Oryza sativa* L.). Plant Sci..

[38] Yang Z.B., Geng X., He C., Zhang F., Wang R., Horst W.J., Ding Z. (2014). TAA1-regulated local auxin biosynthesis in the root-apex transition zone mediates the aluminum-induced inhibition of root growth in *Arabidopsis*. Plant Cell.

[39] Li C., Liu G., Geng X., He C., Quan T., Hayashi K.I., De Smet I., Robert H.S., Ding Z., Yang Z.B. (2021). Local regulation of auxin transport in root-apex transition zone mediates aluminium-induced *Arabidopsis* root-growth inhibition. Plant J..

[40] Jiang D., Du S., Shi J., Xu H., Liu S., Han H., Xu Y., Wang H., Yan M., Huang X. (2025). Glutathione mitigates aluminum toxicity in root-apex transition zone of rice through reducing aluminum absorption and maintaining redox balance. Plant Physiol. Biochem..

[41] Zhang Y., Li Y., de Zeeuw T., Duijts K., Kawa D., Lamers J., Munzert K.S., Li H., Zou Y., Meyer A.J. (2024). Root branching under high salinity requires auxin-independent modulation of LATERAL ORGAN BOUNDARY DOMAIN 16 function. Plant Cell.

[42] Liu J., Otie V., Matsuura A., Junichi K., Irshad M., Zheng Y., Fujimaki H., An P. (2022). Pectin Characteristics Affect Root Growth in Spinach under Salinity. Plants.

[43] Li Y., Wang Y., Tan S., Li Z., Yuan Z., Glanc M., Domjan D., Wang K., Xuan W., Guo Y. (2020). Root Growth Adaptation is Mediated by PYLs ABA Receptor-PP2A Protein Phosphatase Complex. Adv. Sci..

[44] Sun Q., Yamada T., Han Y., Takano T. (2021). Differential responses of *NHX1* and *SOS1* gene expressions to salinity in two *Miscanthus sinensis* anderss. Accessions with different salt tolerance. Phyton.

[45] Tang Y., Du W., Li S., Zhang Z., Zerpa-Catanho D., Song R., Huang H., He X., Kuang X., Liu M. (2026). Multi-omics analysis reveals key genomic insights into salt tolerance mechanisms in *Miscanthus sacchariflorus* and *M. lutarioriparius*. Ind. Crops Prod..

[46] Hosseini S., Moogouei R., Teymoori S., Moogouei R., Borghei M., Latinwo O., Katam K. (2025). Biochemical changes and phytoextraction potential of quinoa and wheat for their resilience to salt stress. Sci. Rep..

[47] Hong C., Zheng B., Guo H., Zhang J. (2019). Salt Stress Induces Shoot Formation and Physiological and Biochemical Changes in *Miscanthus sacchariflorus*. J. Plant Biotechnol. Res..

[48] Lu H., Nkoh J.N., Abdulaha-Al Baquy M., Dong G., Li J., Xu R. (2020). Plants alter surface charge and functional groups of their roots to adapt to acidic soil conditions. Environ. Pollut..

[49] Childress A.E., Elimelech M. (1996). Effect of solution chemistry on the surface charge of polymeric reverse osmosis and nanofiltration membranes. J. Memb. Sci..

[50] Brosse N., Dufour A., Meng X., Sun Q., Ragauskas A. (2012). *Miscanthus*: A fast-growing crop for biofuels and chemicals production. Biofuels Bioprod. Biorefin..

[51] Wang C., Kong Y., Hu R., Zhou G. (2021). *Miscanthus*: A fast-growing crop for environmental remediation and biofuel production. GCB Bioenergy.

